# Papers on Skin Diseases

**Published:** 1867-11

**Authors:** Tilbury Fox

**Affiliations:** Physician to St. John’s Hospital for Skin Diseases


*Papers on Skin Diseases.* By TILBURY FOX, M. D., M. R. C. P., Physician to St. John's Hospital for Skin Diseases.

ON ITCH.—Scabies has undoubtedly been very prevalent amongst us during the last few years; and this has been regarded as a consequence in part of the Crimean war, and in part of the constant non-recognition of the disease and an increase of the over-crowded state of the population. The Americans are experiencing a similar state of things—as a result, they suppose of the late war. Many cases that I see have evidently obtained the disease from ships on their voyage from distant parts of this country. I venture to sketch Scabies from a diagnostic point of view, as the determination of what is and what is not Itch is often difficult.

The effect of the burrowing of the acarus, which is the essential cause of Scabies, is to set up more or less local irritation, according to the state of the patient's nutrition. On a healthy and clean skin no great amount of mischief follows; the acari, however, delight in dirt, and run riot, as it were, on unwholesome surfaces. In the first degree there may be simply those conditions which only necessarily accompany and constitute the mere burrowing of the acarus. The patient complains of itching having all the characters of that of Scabies; but a diagnosis of *pruritus* is erroneously made. The little furrows are unaccompanied by redness, and so delicate that they are overlooked. These cases are rare. The only way in which the papules can be fairly seen is by a side glance with the eye on the level of the skin; they are fine, delicate, slightly elevated, transparent, and contain acari. The suspicious symptom is the itching at night.

Under ordinary circumstances the acarus sets up effusive inflammation, which may reach the stage of papulation or vesiculation or pustulation, the furrow running away, as it were, from the vesicles, which are peculiar, in so far as they are isolated and acuminated.

So, then, there are degrees of Scabies: There is the mere burrowing (the essence of the disease), and certain secondary changes which entirely depend upon the state of the patient's health. Scabies in a filthy pilgrim is very different from that in a clean European; and the Scabies of the upper classes is different from that observed in the ill-fed and badly hygiened of our own people, as represented by the degrees we have spoken of. This fact wants more recognition. It is clear, then, that the amount of eruption depends less upon the number of acari than the state of health, though in sensitive skins a few acari may set up a good deal of erythema and urtication.

In *chronic* Scabies we notice, clinically, two important facts:

1. That the seat of the eruption may shift itself—at one time the hands, perhaps, may be comparatively well, and then a fresh outcrop of vesicles and papules occur.

2. The eruption may vary in intensity: it may diminish in severity, and again become exaggerated according to the hygienic conditions surrounding the patient or the state of his health. In the chronic cases we shall find the remains of the furrows occasionally as rugged lines formed by the shrivelled and broken walls of the furrows. This is very diagnostic of Scabies (chronic).

The acari prefer certain localities to be mentioned directly. There are also spots to which they are frequently specially conveyed, by the child's hand, for instance, to the mamma, by the hand to the penis, the nurse's arms to the buttocks of the child. On the face it seldom occurs in consequence of the influence of the external cold, but in children there are exceptions. In children it is often absent from the hands; beginning about the buttocks, it is seen about the feet especially, and often over the stomach and the well-covered and therefore warm back; when on the face it is occasionally accompanied by sympathetic eczema about the scalp. It is said by some that the acari are only found about the hands, and that the eruptions about the body is entirely sympathetic. Hebra thinks much of the eruption is caused by scratching. No doubt much of the eruption is sympathetic; and although acari are to be found in largest proportion about the hands, yet they are often entirely absent there in the child, and can be detected over other parts of the body.

The following are the diagnostic points of Scabies, but the only really conclusive proof of its existence is the discovery of the furrow and its acarus.

1. Absence of febrile disturbance.

2. Absence of rash from the face and head (this is the rule); its absence from the posterior surface of the arm or body.

3. The seat of the eruption: where the cuticle is thin—as for instance, the interdigital spaces, the anterior surface of the forearm, front of the body below the nipple-level, about the mamma of women, along the front of the penis in men; in the seats of pressure—as for instance, about the groin when trusses are worn, over the ischia, and about the inner line of the wrist, forming a simicircle; in children—the buttocks, the feet, especially the inner line of the sole of the foot, and the palmer surface of the hands.

4. The isolation of the vesicles, and their pointed shape.

5. The *multiformity* of the eruption—namely, the intermingling of papules, vesicles, pustules, scabs, and even small ulcers.

6. The itching at night, and the peculiar linear scratches made with the nails and fringed with dried blood.

7. The cuniculus or furrow—in pustular Scabies few.

8. The evidence of contagion, or the existence of the same sort of disease in one house or family.

It is in children that the greatest mistakes are made, simply from the want of knowing that Scabies does not prefer their hands and arms, but their feet and their buttocks.

With what diseases may Scabies be confounded?

*Lichen.*—But in Lichen the eruption is *uniform.* There are no vesicles or pustules. Lichen occurs on the outer aspect of the forearm. The skin generally is dry, thickened and discolored. And though the back of the hands are sometimes attacked, the interdigital spaces do not suffer.—

The itching is different. There are no cuniculi; no acari of course. It does not occur about the seats of pressure especially. There are no rhagades produced by scratching; and the rash is seen frequently about the face, and often over the back.

*Prurigo.*—In very many cases of Scabies the papules become pruriginous, but not to such a marked degree as in prurigo; and this is only in Scabies a feature superadded. The prurigo of Scabies is seated about the belly and the anterior surface of the forearm; whilst in true prurigo the papules are scattered over the outer aspect of the limbs, over the back, *above* the level of the nipple line, around the neck, in greatest profusion; and about the legs. Moreover, there are no vesicles, though in old standing cases ecthymatous pustules may be developed. But here the origin from Prurigo, and not Scabies, is traceable. Then the skin in Prurigo is unhealthy, the areas of skin enclosed by the natural furrows are exagerated, and a condition of "urtication" results—often times well seen on the back. Pediculi are often present; and the sensation is not itching so much as formication and burning.

*Strophulus* (*or Lichen*) *Pruriginous.*—This is simply Lichen occurring in ill-fed and strumous children, and in consequence the papules are covered at their apices with little points of coagulated blood. This disease lacks altogether the features of Scabies as regards the acarus and its furrow, and the multiform aspect of the secondary eruption; and it is made worse by *the use of sulphur ointment.*

*Eczema.*—This differs entirely from Scabies, in that it is essentially an oozing disease, in which the vesicles are agglomerated (and not isolated and acuminated), forming a patch of greater or less extent; the absence of furrows, &c., of the peculiar itching, of a multiformity in the eruption.

*Sulphur Rash.*—One of the commonest errors is to mistake the artificial rash set up by the use of that villanous compound—the compound sulphur ointment of the old Pharmacopœa—for a continuance and increase of the disease; this has been referred to in a former paper.

*Impetigo Contagiosa*, which I propose to describe at another time especially, mostly begins about the face and head, and is transported hither and thither by the fingers in scratching. It looks like ecthymatous Scabies, but it is not commonly interdigital; the eruption is *uniform*, commencing as isolated vesicles that quickly enlarge into bullæ, with a depressed centre surrounded by a raised collar of blebbed epidermis, the whole being replaced by a light-yellow, flat scab, that looks as if " stuck on " to the part. There are no papules, no furrows, no scratches; it is not interdigital, but attacks the outer aspect of the limbs equally with the inner, the buttock, however, in children and frequently the knees and the feet; the ends of the fingers rather than the palm and interdigits, and this condition is almost always accompanied by eruption on the head and face. Except just at the outset, there is no special itching at night or day either. It is contagious and often epidemic.

*Complicated Scabies.*—Almost any other eruption may commingle with that of Itch. This is very important to bear forcibly in mind; the more we recognize the fact the more likely are we to prevent ourselves being puzzled. We must manage to recognize the co-assemblage of symptoms. Secondary Syphilodermata and Scabies are frequently co-existent. Eczema is very often associated as a sequence, and ought not to offer any difficulty. Scabies in children and congenital syphilis are not unusual. Lichen is sometimes set up and kept a-going by a few acari. *Most cases of lichen urticatus* are dependent upon Scabies. Again, purpura and impetigo contagiosa may be associated with Scabies, the latter frequently. In all these cases we generally have (1) a history of Scabies at the outset; (2) multiformity of eruption, and of course intermingling of the characters of the two coëxistent diseases; (3) the appearance of contagion given to what we hold to be non-contagious disease. For example, a child may seem to catch Lichen from another who perhaps we know has Scabies; the truth being that a few acari have been transplanted, and produced Lichen to such an extent as to have masked the primary mischief, which is only slightly expressed. It is a most excellent rule—one that I adopt myself—to search for Scabies in all cases in which eruptive disease is extensive, and accompanied by much itching at night, especially if it possesses multiformity as one of its features.

*Treatment.*—Scabies never gets well spontaneously. We must treat, 1st, the Scabies itself, killing the acari and their ova; 2nd, the secondary effects; and 3d, the complications. In all cases, *to all papules and vesicles*, the following should be applied: Sulphur, half a drachm; Ammoniated Chloride of Mercury, four grains; Creasote, four drops; Oil of

Camomile, ten drops; and an ounce of lard. This is rubbed in night and morning; the same shirt kept on till the fourth day, when it is changed, and a warm bath given; the ointment to be freely rubbed into the wrists and interdigits specially. In Complicated Scabies, we should treat the Scabies always scrupulously seeking out every suspicious papule, and engraft upon this the plan best suited to the complicating eruption, whatever it may be. We should always remember that in complicated Scabies a small amount of scabies may exist with a good deal of eruption. When the Scabies itself is well, in severe cases a certain period must necessarily elapse before the secondary eruptions can be cured. The process of repair takes time. So we must not persist in the sulphur treatment till all eruption has subsided, in cases of severity. We judge of the cure of Scabies by the itching and by the presence of the vesicles and papules. If we push our sulphur treatment too far, we may produce the state already described as so often mistaken for a continuance and increase of the disease; for this reason the ointment containing the hellebore should not be used.—*London Lancet.*

